# Cannabinoids receptor type 2, CB_2_, expression correlates with human colon cancer progression and predicts patient survival

**DOI:** 10.18632/oncoscience.119

**Published:** 2015-02-09

**Authors:** Esther Martínez-Martínez, Irene Gómez, Paloma Martín, Antonio Sánchez, Laura Román, Eva Tejerina, Félix Bonilla, Antonio García Merino, Antonio García de Herreros, Mariano Provencio, Jose M. García

**Affiliations:** ^1^ Department of Medical Oncology, IIS Puerta de Hierro-Majadahonda, Madrid; ^2^ Department of Pathology, IIS Puerta de Hierro-Majadahonda, Madrid; ^3^ Department of Neuroimmunology, IIS Puerta de Hierro-Majadahonda, Madrid; ^4^ Programa de Recerca en Càncer, IMIM-Hospital del Mar, Barcelona

**Keywords:** Colorectal cancer, prognosis marker, CB2, Disease free survival, overall survival

## Abstract

Many studies have demonstrated that the endocannabinoid system (ECS) is altered in different tumor types, including colon cancer. However, little is known about the role of the ECS in tumor progression. Here we report the correlation between *CB*_2_ expression and pathological data in a series of 175 colorectal cancer patients, as well as the response of the HT29 colon cancer-derived cell line upon *CB*_2_ activation. *CB*_2_ mRNA was detected in 28.6% of samples tested. It was more frequent in N+ patients and predicts disease free survival and overall survival in colon cancer. In positive samples, *CB*_2_ was expressed with great intensity in tumor epithelial cells and correlated with tumor growth. Treatment of HT29 with *CB*_2_ agonist revealed membrane loss of E-cadherin and *SNAIL1* overexpression. A direct correlation between *CB*_2_ and *SNAIL1* expression was also found in human tumors. *CB*_2_ receptor expression is a poor prognostic marker for colon cancer and the activation of this receptor, with non-apoptotic doses of agonists, could be collaborating with disease progression. These results raise the question whether the activation of *CB*_2_ should be considered as anti-tumoral therapy.

## INTRODUCTION

Colorectal cancer (CRC) is the third most common malignancy and the fourth cause of cancer mortality worldwide. The largest fraction of CRC cases is associated with environmental causes rather than inheritable genetic changes. It is unlikely that inflammation initiates sporadic CRC, however chronic inflammation follows tumor development, therefore throughout the progression of the disease a considerable proportion of patients display robust inflammatory infiltration and increased expression of pro-inflammatory cytokines [1]. Consequently, the development and improvement of therapies against the inflammatory microenvironment could be beneficial in the treatment of CRC. To achieve this, the pharmacological modulation of the endocannabinoid system (ECS) should be considered, since the ECS is one of the endogenous mechanisms that control the state of inflammation [2].

Cannabinoids have been used as palliative treatment for chemotherapy in cancer patients, but several studies have proposed the use of cannabinoids as anti-tumoral therapy. The ECS is constituted by the cannabinoid receptors, principally CB_1_ and CB_2_; the endocannabinoids, anandamide (AEA) [3] and 2-arachidonoylglycerol (2-AG) [4,5], and the enzymes that carry out their biosynthesis and degradation. The CB_1_ receptor [6] is mainly present in the central nervous system and mediates the psychotropic effects of exogenous cannabinoids and the analgesic activity. The CB_2_ receptor [7], mainly expressed in peripheral and inflammatory tissues, is responsible for the anti-inflammatory actions of endogenous and exogenous cannabinoids [2]. The ECS suffers a series of adaptive changes in the progression of different diseases, as in cancer development. In general, but with some exceptions specific of tumor type, endocannabinoids and cannabinoid receptor levels in tumor tissues increase regarding their normal counterparts [8]. For colorectal cancer, increases in endocannabinoid levels, down-regulation of CB_1_ and up-regulation of CB_2_ receptor expression have been found [9–11].

Several authors have suggested that cannabinoid agonists have anti-tumoral actions based on *in vitro* studies and with animal models. These anti-tumoral effects are mediated through several mechanisms such as induction of apoptosis in tumor cells, inhibition of proliferation and angiogenesis or anti-metastatic effects through inhibition of tumor cell migration [2,11–14]. However, in some chronic conditions, the alteration of the ECS seems to contribute to the progression and symptoms of the disease. Some studies have found that endocannabinoids and cannabinoid receptor levels are higher in malignant cells or tissues than in non-malignant ones and that there are cases where increased ECS activity correlates with some markers of tumor aggressiveness [14–18]. Since CB_2_ is over-expressed in colon tumors and its activation is not related with psychotropic effects, this receptor could be a good pharmacological target. Nonetheless, it is important to clarify whether CB_2_ collaborates with tumor progression, situation in which inactivation of the receptor could be more appropriate, or whether this over-expression is the response to the inflammatory tumor micro-environment, with the objective of restoring tissue homeostasis, in which case its activation might be desirable.

No study has yet been undertaken that clarifies the involvement of CB_2_ receptor expression in the outcome of colorectal cancer. In this study, we analyzed, in a large series of colorectal cancer patients, the expression of the CB_2_ receptor and its relation with the progression of the disease, in order to shed light on this issue.

## RESULTS

This study was based in a consecutive series of 175 patients diagnosed of CRC at initial stages. Clinical and pathological variables of the series are summarized in Table 1. The median follow-up of the series was 57 months (range of patient follow-up: 1 – 104 months). During the follow-up period, 33.1% recurrence and 32% death occurred. DFS, was 62.29 % (95% CI, 51.18%-73.40%), while OS was, 56.34% (95% CI, 43.35% - 69.33%).

**Table 1:** Correlation between the presence of *CB*_2_ mRNA in tumor samples and clinicopathologic variables

Characteristics	N	Detection of CB_2_ mRNA		
	175	Presence	Absence	*p*
Sex				
Male	108	32 (29.6 %)	76 (70.4%)	0.415
Female	67	18 (26.9%)	49 (73.1%)	
Localization				
Colon	124	33 (26.6%)	91 (73.4%)	0.237
Rectum	51	17 (33.3%)	34 (66.7%)	
Vascular invasion				
Yes	69	20 (29%)	49 (71%)	0.527
No	106	30 (28.3%)	76 (71.7%)	
Polyps	137			
Yes	52	13 (25%)	39 (75%)	0.361
No	85	25 (29.4%)	60 (70.6%)	
Lymph node involvement	137			
Yes	54	21 (38.9%)	33 (61.1%)	0.016
No	83	17 (20.5%)	66 (79.5%)	
Tumor differentiation	175			
Well	63	16 (25.4%)	47 (74.6%)	0.377
Moderate	81	22 (27.2%)	59 (72.8%)	
Poor	31	12 (38.7%)	19 (61.3%)	
Stage				
A	18	5 (27.8%)	13 (72.2%)	0.113
B	92	22 (23.9%)	70 (76.1%)	
C	55	17 (30.9%)	38 (69.1%)	
D	10	6 (60%)	4 (40%)	
Age at diagnosis				
< 71	81	26 (32.1%)	55 (67.9%)	0.214
> 71	94	24 (25.5%)	70 (74.5%)	

### *CB*_2_ mRNA expression in tumor tissue is a poor prognostic factor

CB_2_ receptor mRNA was detected in 50 tumor samples from 175 cases tested (28.6%). This expression correlated with lymph node involvement (LNI) (*p*=0.016) (Table 1).

**Figure 1:**
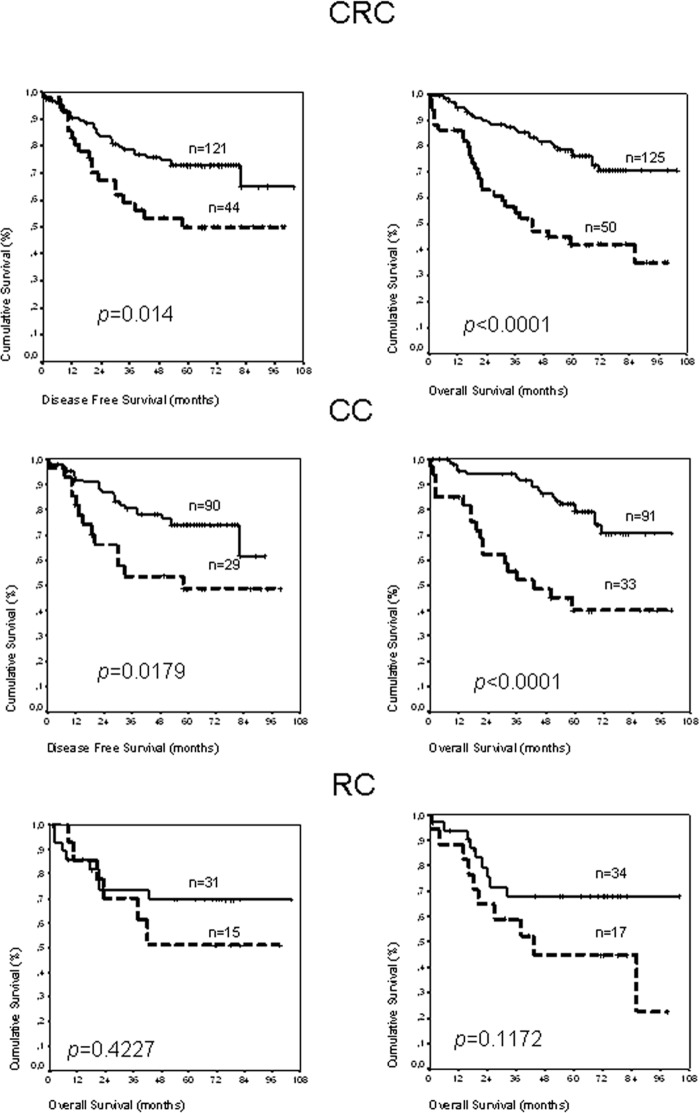
Kaplan-Meier curves and *p* values for DFS (left panels) and OS (right panels) regarding *CB_2_* mRNA expression for the complete colorectal cancer series, CRC; specific for colon cancer series, CC; and for rectal cancer series, RC Patients with tumor in stage IV are not included in the DFS analysis. Number of patients for each cohort is indicated in the graph. Discontinuous line, patients with positive expression of CB_2_. Continuous line, patients with negative expression of CB_2_.

*CB*_2_ receptor expression correlated with both DFS and OS (Figure 1). Concretely, 5-year DFS was 72.84% (95% CI, 64.33%-81.35%) for patients without CB_2_ expression *versus* 49.98% (95% CI, 33.73%-66.2%) for patients with CB_2_ expression (*p* = 0.014). For OS, the differences were even clearer; five-year OS for patients without CB_2_ expression was 76.16 % (95% CI, 67.93%-84.39%) *versus* 41.94% ( 95% CI, 27.37%-56.5%) for patients with CB_2_ expression (*p* < 0.001). Since colon and rectal cancer are considered two different diseases, we carried out the survival analysis in each one of the pathologies. This new analyses showed that CB_2_ mRNA expression is a prognostic factor for colon but not for rectal cancer (Figure 1). In colon cancer patients, the 5-year DFS was 73.83% (95% CI, 64.15%-83.51%) for patients without CB_2_ expression versus 48.68% (95% CI, 28.81%-68.55%) for patients with CB_2_ expression (*p* = 0.018). In contrast, the 5-year DFS for rectal cancer was 69.55% (95% CI, 51.81%-87.29%), for patients without CB_2_ expression, versus 51.14% (95% CI, 22.21%-80.1%) for patients with CB_2_ expression (*p* = 0.42). The results for OS were similar; in colon cancer the five-year OS for patients without CB_2_ expression was 78.98% (95% CI, 69.73%-84.39%) *versus* 40.07% ( 95% CI, 21.8%-58.34%) for patients with CB_2_ expression (*p* < 0.001), while in rectal cancer the five-year OS was 67.88% (95% CI, 50.44%-85.32%) for patients without CB_2_ expression versus 44.82% ( 95% CI, 20.14%-69.5%) for patients with CB_2_ expression (*p* =0.12).

Cox`s regression model confirmed the prognostic value of CB2 expression for both DFS and OS for colon (Tables 2 and 3), but not for rectal cancer (DFS, HR 1.54 (95% CI, 0.53-4.45) (*p* = 0.43), and OS, HR 2.03 (95% CI, 0.82-5.1) (p=0.13)). In addition, LNI, and stage were statistically supported factors in DFS (Table 2) and OS prediction (Table 3) for colon cancer.

**Table 2:** Unadjusted and adjusted analyses of the association between *CB*_2_ expression and disease-free survival of colon cancer patients The blank cells correspond to variables that showed no independent relationship with DFS in the adjusted analysis.

Variable	Category	Unadjusted analysis			Adjusted analysis		
		HR	(95% CI)	*p* Value	HR	(95% CI)	*p* Value
Age at diagnosis	<71 *vs*. >71	0.49	0.24-0.99	0.048			
Sex of patients	Male *vs*. female	1.6	0.78-3.45	0.19			
Lymph node involvement	Yes *vs*. No	4.57	2.29-9.12	<0.001	5.23	2.58-10.6	<0.001
Vascular invasion	Yes *vs*. No	1.47	0.71-3.1	0.3			
Stage	II *vs*. I	1.16	0.34-3.97	0.84			
	III *vs*. I	5.22	1.21-22.64	0.027			
Histological grade	2 *vs*. 1	1.72	0.84-3.52	0.14			
	3 *vs*. 1	0.49	0.11-2.17	0.35			
*CB*_2_ expression	Positive *vs*. negative	2.2	1.07-4.49	0.031	2.77	1.33-5.74	0.006

**Table 3:** Unadjusted and adjusted analyses of the association between *CB*_2_ expression and overall survival of colon cancer patients The blank cells correspond to variables that showed no independent relationship with OS in the adjusted analysis

Variable	Category	Unadjusted analysis			Adjusted analysis		
		HR	(95% CI)	*p* Value	HR	(95% CI)	*p* Value
Age at diagnosis	<71 *vs*. >71	0.57	0.43-1.59	0.57			
Sex of patients	Male *vs*. female	1.22	0.62-2.42	0.56			
Lymph node involvement	Yes *vs*. No	3.72	1.92-7.18	<0.001	4.17	2.12-8.29	<0.001
Vascular invasion	Yes *vs*. No	1.48	0.75-2.93	0.26			
Stage	II *vs*. I	1.4	0.32-6.15	0.66			
	III *vs*. I	4.38	1.004-19.1	0.049			
	IV *vs*. I	60.38	8.1-451.2	<0.001			
Histological grade	2 *vs*. 1	1.43	0.71-2.89	0.31			
	3 *vs*. 1	0.86	0.28-2.6	0.79			
*CB*_2_ expression	Positive *vs*. negative	3.69	1.9-7.2	<0.001	4.2	2.12-8.2	<0.001

The adjusted Cox's regression model showed an independent prognostic value of *CB*_2_ mRNA expression in tumor tissue for DFS, HR 2.77 (95% CI, 1.33-5.74) (*p* = 0.006), and OS, HR 4.2 (95% CI, 2.12-8.2) (*p* < 0.001). LNI maintained its prognostic value in this analysis for both DFS and OS. Because Lymph node involvement and tumor stage are linearly dependent covariates (tumor stages I and II are N−; and tumor stage III and, probably, the vast majority at tumor stage IV are N+), the variable tumor stage was not included in the multivariate analysis.

**Figure 2:**
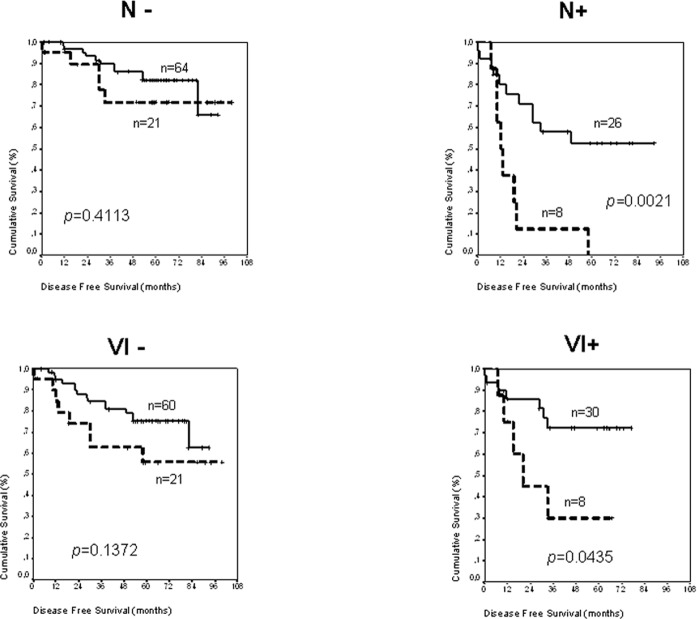
Kaplan-Meier curves and *p* values for DFS regarding *CB_2_* mRNA expression in CC patients without lymph node involvement, N−; patients with lymph node involvement, N+; patients with vascular invasion negative, VI−; and patients with vascular invasion positive, VI+ Patients with tumor in stage IV are not included. Number of patients for each cohort is indicated in the graph. Discontinuous line, patients with positive expression of CB2. Continuous line, patients with negative expression of CB_2_.

Next we analyzed whether CB_2_ expression in colon cancer influence the DFS regarding two prognostic variables, LNI and vascular invasion status. These analyses showed that CB_2_ expression is a prognostic factor only in the group of patients N+ or with vascular invasion (detailed data in Figure 2).

### CB_2_ is up-regulated in tumor epithelial cells from human colon tissues and correlates with tumor growth

**Figure 3:**
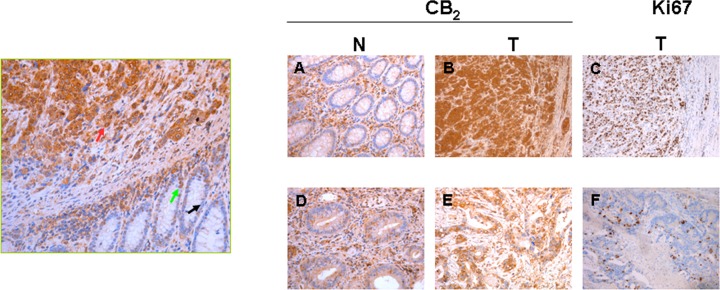
Left panel: Immunohistochemical staining of CB_2_ receptor in human colon from a colorectal cancer patient The representative section contained both normal and tumoral tissue. CB_2_ was expressed with greater intensity in support cells (infiltrating lymphocytes, etc.) and transformed epithelial cells. Normal epithelial cells showed low or negative staining for CB_2_ protein. The intensity of signal is color coded: red arrow indicate high positive staining, green arrow show moderate staining, and black arrow depict negative staining. Right panel: Comparative immunohistochemical analysis of CB_2_, and Ki67 in samples from two patients with high (upper) or low (bottom) expression levels of CB_2_. Normal colonic biopsies (A and D) showed very low staining for CB_2_ in epithelial cells in both cases. Tumoral sections from colorectal cancer patients with high CB_2_ expression (B) showed high Ki67 levels (C). In contrast tumor sample with low CB_2_ expression levels (E) showed low levels of Ki67 (F).

Presence of CB_2_ receptor in epithelial tumor cells was confirmed by immunohistochemistry in tumor samples from 14 patients. In 8 cases the receptor expression was detected in more than 70% of tumor epithelial cells (grade 2); in 3 samples the expression was found between 21-70% of epithelial cells (grade 1); and the remaining 3 cases showed less than 20% of positive stained epithelial tumor cells (grade 0). While in CB_2_-positive tumor epithelial cells staining was observed at high intensity, in normal counterparts staining was weaker and in fewer cells (Figure 3).

Since activation of CB_2_ is related to cell growth inhibition [2], we analyzed Ki-67 levels as a marker of proliferative activity in these patients, who had different CB_2_ receptor expression levels. Unexpectedly, direct correlation between CB_2_ expression levels and the proliferation index was found in these tumor samples (Figure 3). Specifically, 7 of the 8 cases with high proliferation index (Ki-67 ≥ 60%) were classified as grade 2 for CB_2_ expression (87.5%); and only 1 of the 6 cases with low proliferation index (Ki-67 < 60%) was in the grade 2 group for CB_2_ expression (16.7%), *p* = 0.02.

### *Snail1* over-expression in response to CB2 activation

**Figure 4:**
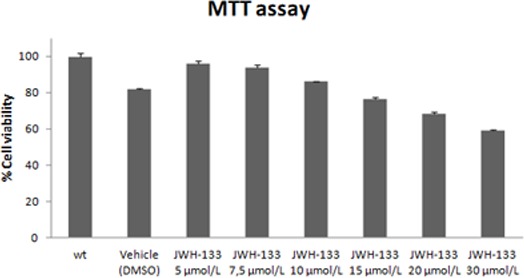
Cytotoxicity of different concentrations of JWH-133 on HT29 cells Cells were incubated in low-FCS medium (0.5% FCS) for 24 hours in the presence of the vehicle (DMSO, 0.06%) or different concentrations of JWH-133 ranging from 5 to 30 μmol/L. Cell viability in JWH-133- and vehicle-treated cells is expressed as mean ± SD of three independent experiments respect no-treated cells (wt, 100%).

**Figure 5:**
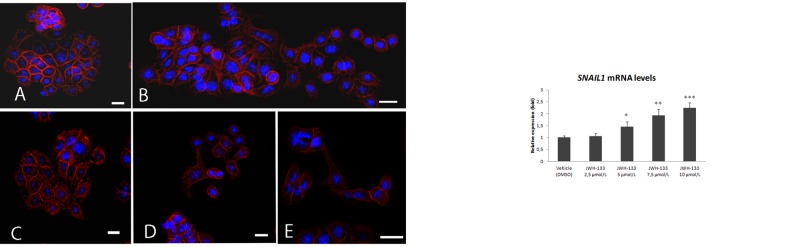
Left panel: Confocal microscopy analysis of E-cadherine HT29 cells were incubated with vehicle (DMSO, 0.02%) (A) or 7.5 μmo/L JWH-133 (B-E) in low-FCS medium (0.5% FCS) for 48h. *Blue*, cell nuclei; *red*, E-cadherine. Right panel: *SNAIL1* expression levels in HT29 cells incubated for 48h with different concentrations of JWH-133. Changes in *SNAIL1* levels are expressed as a fold change compared with the control (DMSO-treated cells). RNA was analyzed by quantitative (real-time) RT-PCR as described in Material and Methods section. Data are expressed as mean±SD of three independent experiments. * *p* < 0,05 ** *p* < 0,005 *** *p* < 0,001.

We analyzed the impact of CB_2_ receptor activation on a colonic epithelial tumor cell line expressing this receptor, HT29 [19], with a specific CB_2_ agonist, JWH-133. To examine this we selected 10 μmol/L as the highest non-apoptotic dose of JWH-133, based on MTT assay (Figure 4). After treatment with increasing doses up to 10 μmol/L for 48h we observed subtle phenotypic changes under the optical microscope, such as differences in cell cluster constitution or in adhesion properties of some cells. These differences led us to analyze possible changes in E-cadherin, the protein responsible for adherent junctions. The immunofluorescence analyses showed a delocalization of E-cadherin from the membrane to the cytoplasm with disperse distribution in several treated cells. The cell clusters, typical of this cell type, were disorganized with zones in the membrane where E-cadherin could barely be found (Figure 5). Moreover, in agonist-treated but not in vehicle-treated cells, there were some cells with a fibroblastic, elongated phenotype (Figure 5).

Based on these observations, we decided to analyze the expression of the *SNAIL1* transcription factor in HT29 cells treated with JWH-133 as described above. A dose-dependent increase of *SNAIL1* was observed after 48 h of treatment (Figure 5).

This correlation was confirmed in 128 CRC samples of the series, from which we had data of *SNAIL1* expression from previous studies[20,21]. We observed direct correlation between the expression of *CB_2_* and *SNAIL1*, in which 72.4% of the tumors expressing *CB_2_* also expressed *SNAIL1*, *versus* 44.4% of the tumors expressing *SNAIL1* when *CB_2_* was not detected, *p* = 0.007.

## DISCUSSION

In recent years, cannabinoids have become a novel therapeutic approach against colon cancer with protective and anti-tumoral effects on colorectal carcinoma cell lines and in animal models of colon cancer [9,11,13,22–25]. In addition, adaptive changes in the ECS have been observed in intestinal biopsies from colon cancer patients, such as increased endocannabinoid levels, down-regulation of CB_1_ and up-regulation of CB_2_ receptor expression [9– 11]. However, there are only a few studies analyzing the involvement of the ECS in colorectal cancer disease.

In this study we verified that CB_2_ is up-regulated in epithelial cells from tumor tissues compared with their normal counterparts. Additionally, the tumors with greater levels of the CB_2_ receptor were those with higher proliferation levels, despite cell-cycle arrest is one of the anti-tumoral mechanisms described for the cannabinoids [2] on “*in vitro”* experiments. The analysis of *CB_2_* mRNA levels in the colon cancer patient series indicated that CB_2_ receptor over-expression is a poor prognostic factor for patients with tumors in advanced stages, patients N+ or patients with tumors that showed vascular invasion. In fact, CB_2_ is more frequently expressed in N+ tumors, suggesting that its expression is related with tumor evolution. However, these patients also have in common that almost of them, contrary to patients with tumors at early stages, are submitted to adjuvant treatments. This consideration opens the possibility that CB_2_ could be a marker for treatment resistance.

Anti-tumoral action of cannabinoids against colon cancer development has been observed with elevated exogenous doses that do not reflect endogenous levels, even in disease [26]. Hart *et al.* described a bimodal action of CB receptor activation, with low (endo)cannabinoid levels being pro-proliferative and high doses of exogenous agonists being anti-proliferative and pro-apoptotic [27]. Our results show that the activation of CB_2_ with non-apoptotic doses of a specific agonist induces an increase in *SNAIL1* expression and phenotypic changes that could be related with the EMT process. We also found positive correlation between *CB_2_* and *SNAIL1* expression in the CRC series, leading us to think that CB_*2*_ receptor is active in CRC tumors. These findings related with EMT process could explain the positive correlation between *CB_2_* expression and LNI, due to the EMT is the first step in the metastasis process.

One of the clinical implications related with the EMT process is the acquisition of therapeutic resistance in those cells where EMT is triggered [28]. This question raises again the possibility that patients with tumors expressing *CB_2_*, expression that correlate with the EMT marker *SNAIL1*, are patients in which the adjuvant treatment is significantly less effective, explaining why *CB_2_* is a prognostic marker only in patients with advanced disease, since this is the group of patients submitted to adjuvant treatment.

In conclusion, our results suggest that CB_2_ is an active protein in CRC cells whose activation collaborates with disease progression. The expression of CB_2_ in tumors is a poor prognostic factor for colon cancer and could be considered as treatment resistance marker. These results shed light about the role of the CB_2_ in the patho-physiology of CC and highlight the importance of the CB_2_ agonist levels that reach to the tumor, because can make the difference between achieve anti-tumor effects or influence in the disease progression.

## METHODS

### Patients and samples

The present study was based on a consecutive series of 175 patients undergoing surgery for CRC. All the experiments carried out in this study complied with current Spanish and European Union laws and the principles outlined in the Declaration of Helsinki. All patients were considered sporadic cases, inasmuch as those with family adenomatous polyposis and clinical criteria for hereditary non-polyposis colorectal cancer (Amsterdam criteria) were excluded. Tumor and normal colon mucosa (taken at least 3 cm from the outer tumor margin) were obtained immediately after surgery, immersed in RNA*later*^TM^ (Ambion Inc, Austin, Texas), snap-frozen in liquid nitrogen and stored at −80°C until processing.

### Reagents and Drugs

JWH-133 was purchased from Tocris Cookson (Bristol, UK). The drug was dissolved in dimethylsulfoxide (DMSO).

Antibodies for immunohistochemistry and confocal microscopy were purchased as follows: mouse monoclonal anti-CB_2_ (clone 352114) was from R&D systems (Minneapolis, USA), mouse monoclonal anti-human Ki-67 (clone MIB-1) was from Dako (Glostrup, Denmark) and mouse monoclonal anti-E-cadherin (clone 36/E-cadherin) came from BD Transduction Laboratories^TM^.

The MTT Cell Proliferation Assay Kit was purchased from Cayman Chemical (Ann Arbor, MI).

### Immunohistochemical analysis

Immunohistochemical staining for Ki-67 and CB_2_ was performed in 14 CRC samples. 4-μm-thick sections were cut from formalin-fixed and paraffin-embedded tissue blocks. Ki-67 expression was analyzed with the clone MIB-1, at 1/50 working dilution. CB_2_ expression was analyzed with a mouse monoclonal antibody at 1/50 working dilution. The staining procedure for Ki-67 and CB_2_ was performed on the Dako Cytomation Autostainer and automated Leica Bond Max system (Leica Microsystems, Germany), respectively. The slides were counterstained with Mayer's hematoxylin, dehydrated and mounted with DePex (BDH, Poole, Dorset, UK). Negative control slides were not exposed to the primary antibody and were incubated in PBS and then processed under the same conditions as the test slides.

CB_2_ staining in tumor samples was recorded through a three-grade system based on the percentage of tumor epithelial cells stained: grade 0 = 1% to 20%, grade 1 = 21% to 70% and grade 2 = more than 70% [11]. Samples with ≥60% of nuclei stained were classified as Ki-67 high [29].

### Clinico-pathological parameters of the patients

The parameters obtained from the medical records of the 175 patients were: age, tumor location, lymph node involvement (LNI) (evaluated by optical microscopy), pathological stage (assessed by the tumor-node-metastases classification), tumor histological grade and the presence of vascular invasion in tumors, Table 1.

Patients' clinical follow-up after surgery and diagnosis was based on periodic visits and clinical, biochemical and imaging techniques. Ultrasonic study was performed when liver function was impaired. Overall and Disease-Free Survival were defined as the period of time from diagnosis to death and the interval between diagnosis and first recurrence, respectively.

Colon cancer patients did not receive neo-adjuvant chemotherapy (CT). Patients with rectal carcinoma who had received preoperative treatment with CT and radiotherapy or radiotherapy alone were excluded. Adjuvant treatment based on oxaliplatin (FOLFOX6, leucovorin 400 mg/m_2_ IV on day 1 as a 2-hour infusion, followed by 5-fluorouracil bolus of 400 mg/m_2_ IV on day 1, followed by 2,400 mg/m_2_ IV 46-hour infusion and oxaliplatin 100 mg/m_2_ IV as a 2-hour infusion on day 1) was administered to 52 stage-III patients (31 colon cancer and 21 rectal cancer), and to 11 stage-II colon cancer patients without medical contra-indications who gave their written informed consent. Radiotherapy was also administered to 49 rectal tumor cases.

### Real Time RT-PCR

*SDHA* (Succinate Dehydrogenase Complex subunit A) mRNA expression was used as reference gene. *SDHA* mRNA in all human samples included in this study was detected before cycle 30 of amplification. *CB_2_* expression was valued in tumor tissues as presence or absence. *SNAIL1* mRNA expression in cell lines was referenced to *SDHA* mRNA.

The gene expression analysis was performed in duplicate. The primers used were: *SDHA*-5′TGGGAACAAGAGGGCATCTG 3′ forward (F) and 5′CCACCACTG-CATCAAATTCATG 3′ reverse (R); *CB_2_*-5′AGCCACCCACAACACAACC 3′ forward (F) and 5′GAGCCATTGGCTATCTCTGTC 3′ reverse (R); *SNAIL1*-5′CAC-TATGCCGCGCTCTTTC 3′ forward (F) and 5′GGTCGTAGGGCTGCTGGAA 3′ reverse (R) The annealing temperature was 59°C for *SDHA* and *CB_2_* and 68°C for *SNAIL1*. At the end of the PCR cycles, melting curve analyses were performed to confirm the generation of the specific expected PCR product. The PCR products were sequenced in an ABI Prism_TM_ 377 DNA sequencer apparatus (PE Applied Biosystems). For the synthesis of cDNA, 400 ng of total RNA was retro-transcribed, using the Gold RNA PCR Core Kit (PE Biosystems, Foster City, CA). Real-time PCR was performed in a Light-Cycler apparatus (Roche Diagnostics, Mannheim, Germany), using the LightCycler-FastStart_PLUS_ DNA Master SYBR Green I Kit (Roche Diagnostics, Mannheim, Germany).

### Cell culture and drug treatments

*In vitro* experiments were performed with the colon carcinoma cell line HT29, purchased from the American Type Culture Collection (ATCC). Cells were cultured in Dulbecco's modified Eagle medium (DMEM) (Gibco Life Technologies, Gergy-Pontoise, France), containing 10% heat-inactivated fetal calf serum (FCS), 2mM L-glutamine, penicillin (100 U/mL), streptomycin (100 ng/mL) and fungizone (0.25 μg/mL) at 37°C in a 5% CO2-humidified atmosphere.

Cell viability was determined by MTT assay. 1×10_4_ cells/well were seeded in 96-well plates in DMEM 10% FCS. During the treatment with CB_2_ agonists, the medium was replaced by low-FCS medium (0.5% FCS) and cells were incubated for 24h with the vehicle or different concentrations of agonists. The MTT assay was performed according to the manufacturer's protocol.

For drug response assays, cells were grown to 60-80% confluence in 6-well plates. Cells were treated with drug vehicle or different concentrations of agonists in low-FCS medium for 48 hours with drug refreshing every 24 hours. *SDHA* and *SNAIL1* expression levels were measured by real-time PCR.

### Confocal microscopy

HT29 cells were grown in 6-well culture clusters (Nunc, NY, USA) and treated with JWH-133 for 48h. Then, cells were fixed with Methanol for 10 minutes, washed with PBS, incubated in 50 mM NH_4_Cl and blocked with 5% BSA to reduce non-specific protein binding. Cells were incubated with Anti E-Cadherin (1/25) overnight at 4°C, washed with PBS and followed with Alexa Fluor 546 anti-mouse (Invitrogen Life Technologies, 1/1000) for 45 minutes at room temperature. Nuclei were stained with Topro-3 (Invitrogen Life Technologies 1/1000) for 15 minutes and cells were visualized with inverted Microscopy. Images of the specimens were collected with a TCS SP5 confocal microscope (Leica Microsystems, Wetzlar, Germany), equipped with 10×0.22 and at an optical zoom of 3. Z-series images were obtained through the collection of serial, confocal sections at 1-μm intervals.

### Statistical analysis

CB_2_ expression was contrasted with Ki-67, *SNAIL1* expression data and with clinico-pathological parameters by the χ_2_ test. Statistical significance for *SNAIL1* expression in treated cell lines was assessed by two-tailed unpaired Student's *t* test. Differences were considered statistically significant when *p* < 0.05.

DFS analysis did not include patients at pathological stage IV. The relationship between the cumulative probability of OS and DFS, as well as analyzed predictors, was calculated with the Kaplan-Meier method, while significant differences between curves were evaluated with Mantel's log-rank test. To identify factors that might be of independent significance in influencing OS and DFS, multivariate analysis (Cox proportional risk regression model) was applied. The model's basic assumptions (proportional hazards) were evaluated. In all statistical tests, two-tailed *p* values ≤ 0.05 were considered statistically significant. Statistical analyses employed the SPSS 13.0 statistical software (SPSS Inc., Chicago, IL).

## Abbreviations

ECS: Endocannabinoid system
CRC: Colorectal cancer
DFS: disease-free survival
OS: overallsurvival
LNI: lymph node involvement
EMT: epithelialmesenchymal transition
CB2: Cannabinoid receptor type 2.
